# Knee Swing Phase Flexion Resistance Affects Several Key Features of Leg Swing Important to Safe Transfemoral Prosthetic Gait

**DOI:** 10.1109/TNSRE.2021.3082459

**Published:** 2021-06-03

**Authors:** Jenny A. Kent, V. N. Murthy Arelekatti, Nina T. Petelina, W. Brett Johnson, John T. Brinkmann, Amos G. Winter, Matthew J. Major

**Affiliations:** Department of Physical Medicine and Rehabilitation, Feinberg School of Medicine, Northwestern University, Chicago, IL 60611 USA; Global Engineering and Research Laboratory, Department of Mechanical Engineering, Massachusetts Institute of Technology, Cambridge, MA 02139 USA.; Global Engineering and Research Laboratory, Department of Mechanical Engineering, Massachusetts Institute of Technology, Cambridge, MA 02139 USA.; Global Engineering and Research Laboratory, Department of Mechanical Engineering, Massachusetts Institute of Technology, Cambridge, MA 02139 USA.; Department of Physical Medicine and Rehabilitation, Feinberg School of Medicine, Northwestern University, Chicago, IL 60611 USA; Global Engineering and Research Laboratory, Department of Mechanical Engineering, Massachusetts Institute of Technology, Cambridge, MA 02139 USA.; Department of Physical Medicine and Rehabilitation, Feinberg School of Medicine, Northwestern University, Chicago, IL 60611 USA; McCormick School of Engineering, Northwestern University, Chicago, IL 60208 USA, and also with the Jesse Brown VA Medical Center, Chicago, IL 60612 USA

**Keywords:** Design optimization, gait, prosthetics, swing phase control, transfemoral amputation

## Abstract

We systematically investigate *in-vivo* the effect of increasing prosthetic knee flexion damping on key features of the swing phase of individuals with transfemoral amputation during walking. Five experienced prosthesis users walked using a prototype device in a motion capture laboratory. A range of interchangeable hydraulic rotary dampers was used to progressively modify swing phase flexion resistance in isolation. Toe clearance (TC; vertical distance toe to floor), effective leg length (ELL; distance hip to toe), and knee flexion angle during swing phase were computed, alongside the sensitivities of vertical toe position to angular displacements at the hip, knee and ankle. Key features of these profiles were compared across 5 damping conditions. With higher damping, knee extension occurred earlier in swing phase, promoting greater symmetry. However, with implications for toe catch, minimum TC reduced, and minimum TC and maximum ELL occurred earlier; temporally closer to mid-swing, when the limb must pass the stance limb. Further, TC became less sensitive to changes in hip flexion, suggesting a lesser ability to control toe clearance without employing proximal or contralateralcompensations.Thereisatrade-offbetweenkeyfeaturesrelated to gait safety when selecting an appropriate resistance for a mechanical prosthetic knee. In addition to highlighting broader implications surrounding swing phase damping selection for the optimization of mechanical knees, this work reveals design considerations that may be of utility in the formulation of control strategies for computerized devices.

## I. INTRODUCTION

LOWER limb amputation at the transfemoral level (above the knee) presents a challenge for the design and configuration of prosthetic components aimed to restore mobility, due to the loss of the natural knee joint. During the swing phase of gait, i.e., the period between the foot leaving the ground (*foot off*) and subsequent *foot contact*, the knee exhibits the largest angular range of motion of any of the lower limb joints, and its movement trajectory incorporates a directional shift from flexing to extending. Sufficient knee flexion in mid-swing is necessary to ensure adequate clearance between the foot and walking surface, to prevent toe catch [1]. Conversely, full or near-full *extension* at terminal swing promotes an extension moment on loading; precluding buckling and thus permitting a safe transition from swing to the stance phase of gait [2], i.e., the period during which the foot is in contact with the ground. The appropriate timing of these features of movement within a given stride is critical in achieving safe and efficient walking. As the action of a conventional prosthetic knee joint is not under direct volitional control of the user (although progress is being made in this direction [3], [4]), this behavior must be realized through the design and configuration of the device itself.

The majority of individuals with transfemoral amputation rely upon a purely mechanical knee joint during their daily life as the cost of microprocessor-controlled knees (MPKs) remains prohibitive for many users worldwide [5]. During walking, the behavior of a mechanical knee, in the absence of imposed control algorithms, is largely dictated by its physical and mechanical properties under the influence of gravity. A knee with a simple hinge under negligible frictional forces will swing as a pendulum, with oscillation period determined by the length of the lower leg; unadaptive to changes in cadence or walking speed [2]. Should excessive knee flexion (or excessive *heel rise*, in clinical terms) occur during early swing due to the double pendulum effect, the user must wait longer for the limb to reach the requisite extended position prior to foot contact, to prevent buckling when loaded [6].

The extent to which a passive knee joint flexes during the critical early swing period at a given walking speed can be manipulated by adjusting the resistance to motion via mechanical friction or fluid damping. Fluid actuators (e.g., hydraulic and/or pneumatic) provide resistance that is proportional to angular velocity or its square, and have been increasingly incorporated into prosthetic designs as they can accommodate a modest range of walking speeds with no requirement for explicit additional adjustment of the device by user or prosthetist [7], [8]. With such a device, selection of an appropriate level of baseline swing-phase knee flexion damping (KFD_SW_*)* is guided by these two competing requirements for knee motion; i.e., the flexion of the knee in early swing must be appropriate in both magnitude and timing to (i) shorten the limb to permit it to pass the stance limb and to (ii) facilitate full extension of the limb prior to foot contact to permit safe loading; and both without substantial movement deviation or disruption to stride timing. Whilst a trade-off seems inevitable, there is little empirical evidence demonstrating the relative effect of changes in KFD_SW_ on these two basic, yet fundamental requirements; although their importance is readily acknowledged (e.g., [1], [9]), they are seldom measured.

Although passive knee behavior may be modelled and predicted, it is unclear how changes in KFD_SW_ in isolation are accommodated holistically during the swing phase. The trajectory of the foot is dependent on the coordination of rotations at the hip in addition to the knee, and also subject to contralateral and more proximal movements that increase the vertical position of the foot during the swing phase, e.g., contralateral plantarflexion during ipsilateral swing phase (‘vaulting’) or pelvic obliquity/hip hike [10]. Such modifications to behavior have the potential to result in outcomes that deviate from those predicted based on mechanics alone, with implications for both gait safety and efficiency. There have been several studies examining the efficacy of fluid damping mechanisms *in-vivo* by comparing performance when using different prosthetic components (e.g., [1], [6], see [11] for a recent review). However, the effect of KFD_SW_ magnitude itself is not isolated, due to differences in multiple concurrent prosthetic design factors, including geometry, mass, alignment, stance phase resistance mechanism, and presence of microprocessor control.

The objective of this study was to systematically explore the effect of adjusting KFD_SW_ on swing phase dynamics during locomotion. Using a unique prototype knee that permits adjustments in KFD_SW_ to be made in isolation of other factors, we specifically aimed to provide a fuller understanding of the implications surrounding these two competing functional requirements for knee motion during swing: (i) foot clearance, and (ii) knee extension in preparation for foot contact. In accordance with purely physical mechanics, we anticipated that increments in KFD_SW_ would lead to measurable decrements in peak knee flexion in early swing, reducing the extent to which the limb shortens to pass the stance limb. We first sought to verify this prediction through the examination of the prosthetic side *effective leg length*; an analog of knee flexion that is of functional relevance given these device requirements, defined as the distance from hip to toe. Second, we aimed to explore the consequences of these changes in motion on swing phase dynamics in the presence of user compensations, through the following research questions:

1. What is the effect of KFD_SW_ on *toe clearance*; the proximity of the toe to the ground, during swing?
2. How does KFD_SW_ affect the *relative timing* of key instances in the swing phase of the prosthetic side?
  Finally, given that the hip is the only natural, and therefore volitionally-controlled, joint of the affected limb, we posed the following question:
3. Could KFD_SW_ affect the extent to which swing phase toe clearance may be *volitionally regulated* through the manipulation of flexion or extension of the ipsilateral hip?

In addition to informing passive device configuration, this objective information may guide algorithm optimization for future microprocessor-controlled units, and ankle-knee designs, to facilitate safe and efficient ambulation with a transfemoral prosthesis.

## II. METHODS

The Jesse Brown VA Medical Center Institutional Review Board granted ethical approval for this study, and five individuals with a unilateral transfemoral amputation (2M, 3F; 47.4 ± 18.2 yrs, 1.60 ± 0.10 m, 73.6 ± 24.8 kg; Table I) provided written informed consent to participate.

### A. Device and Device Configuration

All fitting and clinical alignment was performed by the same experienced prosthetist. Participants were fitted with a passive, single-axis prototype knee with a prototype foot [12], maintaining their existing socket and suspension. The knee unit, described in more detail in [13]–[15], permits adjustment of KFD_SW_ via rotary hydraulic dampers attached to the medial or lateral aspect of the device. The resistance provided is linearly proportional to angular velocity. Damping is restricted to the flexion phase of swing by means of a one-way clutch, with an extension bumper to minimize terminal impact effects. With minimal increase in mass (0.15–0.22 kg), predicted by prior simulations to have negligible impact on hip dynamics [16], a range of fluid damping settings from 0 Nm/rad/s (mechanical friction only; condition A) to ~1.80 Nm/rad/s, in discrete increments, could be tested. This spanned the range of damping coefficients predicted from able bodied knee dynamics (knee flexion angles, angular velocities, moments) over walking speeds 0.6–1.3 m/s, accounting for differences in inertial properties (see [14], [17]). Five to six damping settings were selected for each participant to exceed the range of optimal damping conditions predicted to replicate physiological knee motion, based on individual body mass (see [14], [17]). The foot was an energy storage and return design with a flexible cantilever beam forefoot and ankle joint with torsional stiffness provided by interchangeable springs. Ankle stiffness and forefoot length were customized for each participant based on height, body mass and intact foot length (refer to [12] for description and validation).

### B. Familiarization

Four of five participants completed testing on two days, with data for the present study collected on the second. Due to time constraints, the fifth participant attended only once but was provided with additional familiarization time during the single test session. In all sessions, following alignment and instruction, each participant completed a process of familiarization with the prosthesis in condition A (no fluid damping) within parallel bars, progressing to unassisted walking when confident in their ability to safely walk without aids. This process lasted approximately 5–10 minutes and continued until the participant stated that they were comfortable to proceed. In the initial familiarization session (P1-P4) the participant then walked on a treadmill equipped with a harness and handrails (T170, h/p/cosmos sports & medical Gmbh, Nu*β*dorf, Traunstein, Germany) for 1–2 minutes with no damping, then completed an additional 2–3 minutes of familiarization in each damping condition. In the test session, to permit the participant to experience a change in damping prior to data collection, a damper approximately in the middle of the range determined for the individual was selected, and the familiarization process was repeated. Participant 5 completed an additional 1–2 minutes of treadmill familiarization in two conditions prior to data collection.

### C. Data Collection

Participants wore their own footwear on their unimpaired side and comfortable clothing. The prototype foot [12] was designed to be used without a shoe, and the prosthetic limb was configured to account for this. Reflective markers were placed on the pelvis and bilaterally on the feet, lower legs and thighs (see Supplementary Information file S1). Additional markers on the trunk, elbows and wrists were attached but not utilized for the purposes of this analysis. On the prosthesis, markers were placed at the single axis center of rotation of the knee and at the axis of rotation of the prototype ankle.

Assessments were conducted in order of increasing damping for safety purposes because this steady progression was anticipated to be less destabilizing with limited acclimation time. Participants were not informed what aspect of the prosthesis was specifically being adjusted. The process of data collection was identical for each damping condition. Following configuration and prior to the capture of walking data, participants completed at least two traverses of the laboratory floor for familiarization and a standing trial was collected for model calibration purposes. The participant was then asked to perform repeated 10 m traverses of the laboratory “at a comfortable speed” during which kinematic data were collected at 120 Hz in Cortex software using a 12-camera motion capture system (both Motion Analysis Corp., Santa Rosa, CA, USA). Synchronous kinetic data, used for gait cycle event detection purposes in the present study only, were acquired at 960 Hz from floor-embedded force plates (AMTI, Watertown, MA, USA). The collection for each condition was terminated when data for five strides that included a clean force plate contact had been attained on each leg, which was accomplished within approximately 8–20 trials. Participants were provided 5–10 minutes of seated rest between each condition, whilst the next damping condition was configured.

### D. Data Processing

Data were labelled and filtered with a 4th order bidirectional 6 Hz low pass Butterworth filter in Cortex software (v7, Motion Analysis Corp., Santa Rosa, CA), and then exported to Visual 3D (C-motion Inc., Bethesda, MD, US) where a pelvis, trunk and lower limb model (see Supplementary Information file S1) was applied. To estimate toe clearance, an additional virtual toe landmark (vTOE) defined at a location estimating the distal endpoint of the prosthetic foot was required. The heel and toe marker locations were first projected onto the plane of the laboratory floor within the standing calibration file. The vTOE landmark was then defined on an axis passing through these projected landmarks, at a distance equal to the measured foot length from the centroid of the projected heel marker, accounting for marker radius. The position of this landmark was then computed during the swing phase of gait during the walking trials based on its relation to the coordinates of the toe, heel and metatarsal head markers. This approximation technique avoided the requirement for additional physical motion capture markers and assumed that, in the absence of volitional control, the foot behaved as a rigid body once unloaded.

Foot off events were identified based on the transition from above to below a vertical force plate threshold of 5 N. The ipsilateral foot contact following each force plate contact, required to define the end of swing phase, was identified based on the vertical and anterior-posterior position of the proximal end of the foot segment (approximate ankle joint center), and verified manually by observation of the skeleton avatar. A mid-swing event (MSWt) was defined for each stride as the frame closest to the time point at which the ankle joint center of the swing limb passed that of the stance limb in the direction of walking, aligned with the laboratory coordinate system.

**Fig. 1.**
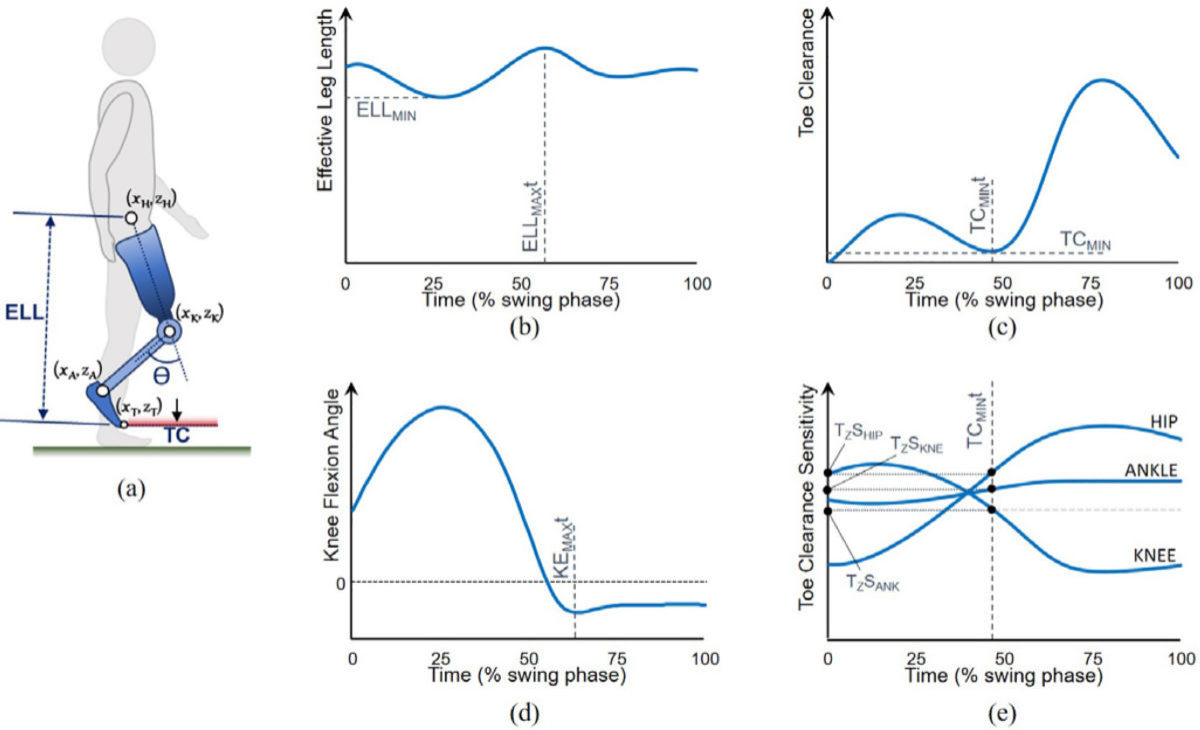
Swing phase variables. (a) Definition of primary variables: Effective Leg Length (ELL), and Toe clearance (TC) with knee flexion angle (*θ*). x and z with subscripts HIP, KNE, ANK and TOE refer to anterior-posterior (x) and vertical (z) coordinates of the hip, knee, ankle, and toe respectively. (b)-(e) Illustrative time-normalized profiles during the swing phase of gait. ELL_MIN_ – minimum effective leg length, TC_MIN_ – minimum toe clearance, ELL_MAX_t, TC_MIN_t, KE_MAX_t – timings of maximum leg length, minimum toe clearance and maximum knee extension respectively. TZS_ANK_, _KNE_, _HIP_ – sensitivity of toe height to changes in ankle, knee, and hip rotation, respectively, at the time of minimum toe clearance.

The effective leg length, i.e., the distance between the vTOE and the estimated hip joint center [18] and toe clearance, i.e., the vertical distance between the estimated vTOE and the ground [18] (Fig. 1a), were computed across each swing phase (foot off to ipsilateral foot contact). Sagittal plane knee angle was computed as the relationship between lower leg and thigh segments about the medial-lateral axis of the thigh (Fig. 1a). In addition, the sensitivity of the vertical position of vTOE to changes in knee, hip and ankle flexion was computed across each swing phase using the method of Moosabhoy & Gard [18], using (1)-(3) based on partial derivatives of toe height (zTOE) with respect to hip flexion angle, *α*; knee flexion angle *β* and ankle dorsiflexion angle, *γ* (Fig. 1a), where x_TOE_, x_HIP_, x_KNE_, and x_ANK_ are horizontal coordinates of the toe, hip, knee and ankle respectively (see [18] for derivation).

(1) $$\frac{\partial z_{TOE}}{\partial \alpha }=x_{TOE}-x_{HIP}$$

(2) $$\frac{\partial z_{TOE}}{\partial \beta }=x_{KNE}-x_{TOE}$$

(3) $$\frac{\partial z_{TOE}}{\partial \gamma }=x_{TOE}-x_{ANK}$$

### E. Data Extraction

Effective leg length, toe clearance, sagittal plane knee angle and joint sensitivities for the hip, knee and ankle were plotted as a function of time, normalized to swing phase, for each participant and each condition. For each identified swing phase, the following variables were then computed:

- Minimum effective leg length, ELL_MIN_; the shortest vertical distance between the estimated hip joint centers and vTOE positions (Fig. 1b).
- Minimum toe clearance, TC_MIN_; the minimum vertical distance between vTOE and floor (note that smaller toe clearance indicates the toe is nearer the ground - logically opposite in direction to effective leg length) (Fig. 1c).
- Timing of maximum knee extension in terminal swing, KE_MAX_t, expressed as a percentage of the swing phase (Fig. 1d).
- Timing of maximum effective leg length in swing phase, ELL_MAX_t, and with respect to timing of mid-swing (positive values indicate ELL_MAX_t follows MSWt); expressed as a percentage of the swing phase (Fig. 1b). Note, ELL_MAX_t is related to, but not coincident with KE_MAX_t due to limb geometry, and whereas the latter directly probes the requirement for knee flexion on loading (requirement ii), ELL_MAX_t better aids the analysis of foot clearance (requirement i).
- Timing of minimum toe clearance in swing phase, TC_MIN_t, and with respect to timing of mid-swing (positive values indicate TC_MIN_t follows MSWt); expressed as a percentage of the swing phase (Fig. 1c).
- Swing time symmetry (STS), expressed by prosthetic side swing phase duration as a percentage of prosthetic side plus sound side swing phase durations, where 50% would indicate perfect symmetry, and values *>*50% indicate a greater swing duration on the prosthetic side.
- Sensitivity of vertical toe position, TZS, to ankle, knee, and hip flexion at the timing of minimum toe clearance, in m/rad (Fig. 1e). Positive (vs. negative) TZS values imply that increasing flexion at the joint would act to increase (vs. decrease) toe clearance. Further, the magnitude of TZS indicates the extent to which toe clearance can be influenced by the angular rotations of each joint; greater absolute TZS values indicate a greater change in the vertical position of the toe would be achieved per radian of angular rotation. Thus, when looking specifically at the natural hip joint on the affected side, the magnitude of TZS provides a proxy for the control capacity of the joint.

Finally, three common movement compensations were explored for each participant in each damping setting to elucidate deviations that may have influenced the variables under investigation. Walking speed was computed from the average velocity of the body center of mass across the traverse. The vertical position of the hip joint center at TC_MIN_t was extracted as an illustration of compensations used to increase the proximal limb position as an aid to clearance (i.e., contralateral midstance plantar flexion, and/or ‘vaulting’, and ipsilateral hip raise, or ‘hip hike’). The lateral distance between the hip joint center and ankle joint center was extracted at TC_MIN_t as a proxy for limb ‘circumduction’, i.e., lateral deviation of the limb induced by non-sagittal plane rotation at the hip.

For each variable, the average (mean) of 5 strides was computed, for each participant and condition, with the exception of walking speed, for which the first five complete traverses used in the analysis were included. Line graphs were generated for further qualitative analysis. Damping coefficients approximated from previous bench testing (see [19], [20]) were tabulated for each participant based on the KFD_SW_ conditions tested. In order to evaluate the strength and order of trends observed, the relationship between KFD_SW_ and each variable (a)-(g) were assessed at an individual level by linear and 2nd order polynomial least squares regressions. R^2^ values measuring the amount of variance accounted for by each model fit were averaged across the five participants.

Hip and knee angles at TC_MIN_t have been provided in Supplementary Information file S2 to further aid the reader in interpretation of the results.

## III. RESULTS

Participants 1–3 completed trials in six damping conditions and 4–5 completed only five; the final was omitted as it was considered unsafe to increase the damping further due to excessive toe catch observed at the fifth condition. Further, during bench testing it was determined that two dampers were not performing as anticipated, leading to the exclusion of one condition for each of participants 1–3. Participant characteristics and the final KFD_SW_ settings for each participant are provided in Table I. All participants habitually walked with energy storage and return-type feet. Four (P2-P5) customarily used microprocessor-controlled knees. P1 used a single axis hydraulic knee (Mauch SNS, Össur, Reykjavik, Iceland).

**Fig. 2.**
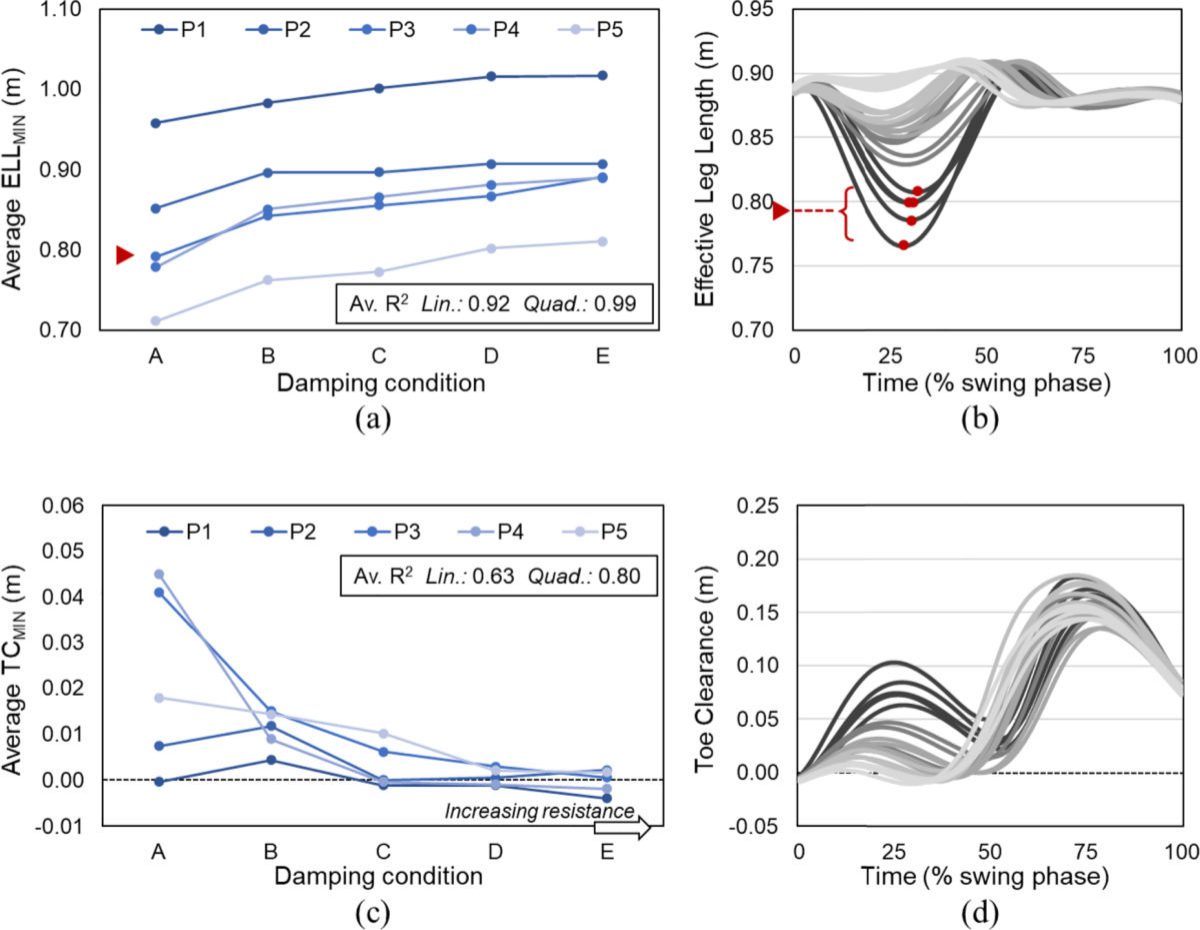
Effect of swing phase knee flexion damping on effective leg length and toe clearance. A-E in order of increasing damping. Left panel shows average minimum values of effective leg length [(a); ELL_MIN_] and toe clearance [(c); TC_MIN_] with each line representing a different participant (P1-P5) and each point the average (mean) of 5 trials. Note, negative TC_MIN_ values attained as a result of toe approximation. Right panel [(b), (d)] shows representative time series data from a single participant (P3) as a function of time, normalized to swing phase; shading from dark to light in order of increasing damping. Red arrows illustrate derivation of a single averaged point from individual data. Av. R^2^ indicates linear (lin) and quadratic (quad) goodness-of-fit, computed on an individual basis with respect to KFD_SW_ and averaged across participants.

**Fig. 3.**
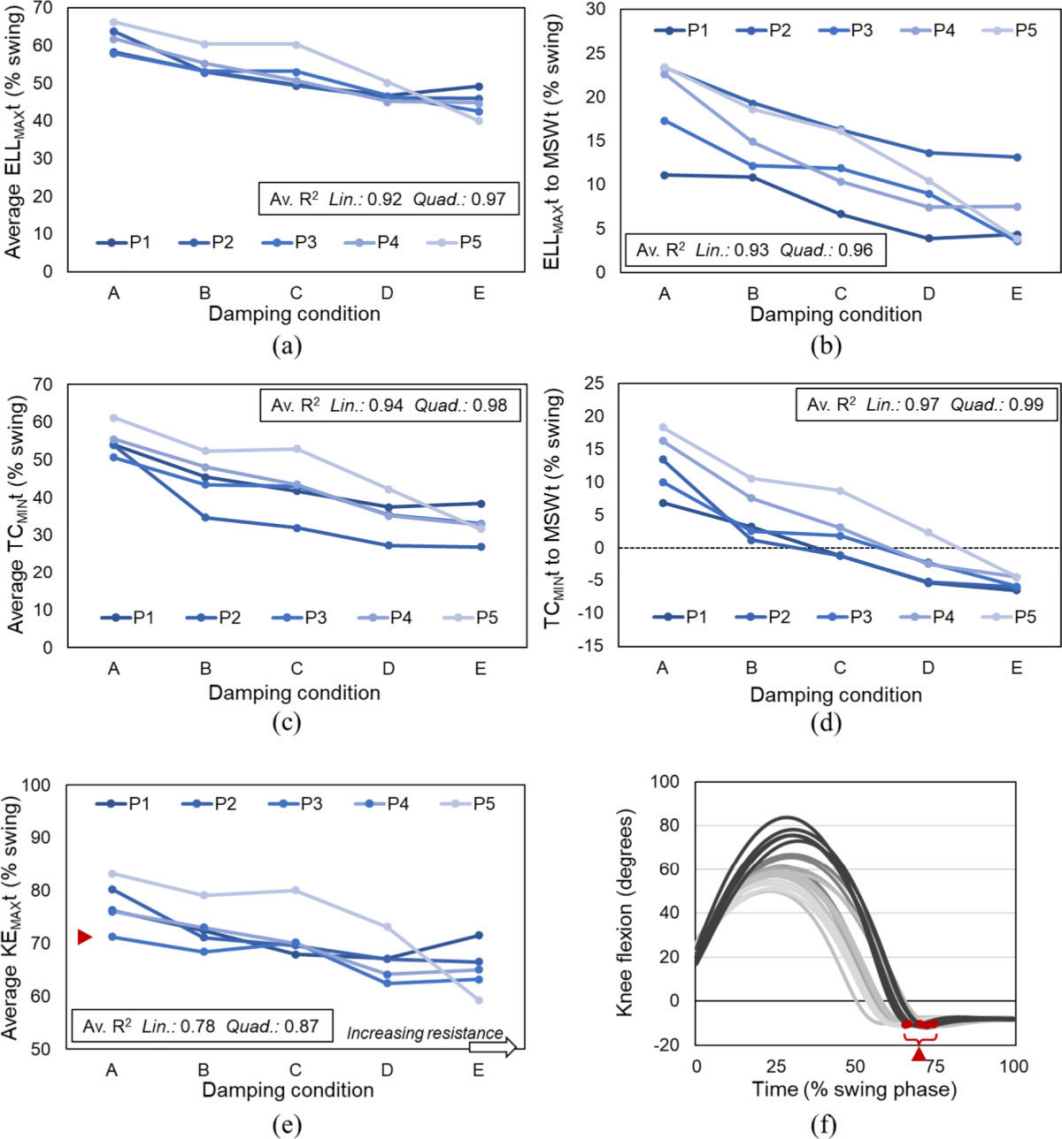
Effect of swing phase knee flexion damping, KFD_SW_, on swing phase event timing. Effective leg length [ELL_MAX_t: (a),(b)], toe clearance (TC_MIN_t; c,d) and knee extension [KE_MAX_t; (e),(f)]. MSWt – time of mid-swing. (a)–(e) – average (mean) of 5 strides for each participant (P1-P5) at increasing KFD_SW_ levels (A-E). Av, R^2^ indicates linear (lin) and quadratic (quad) goodness-of-fit, computed on an individual basis with respect to KFD_SW_ and averaged across participants. (f) – representative time series data from a single participant (P3) as a function of time, normalized to swing phase; shading from dark to light in order of increasing damping. Red arrows illustrate derivation of a single averaged point from individual data.

**Fig. 4.**
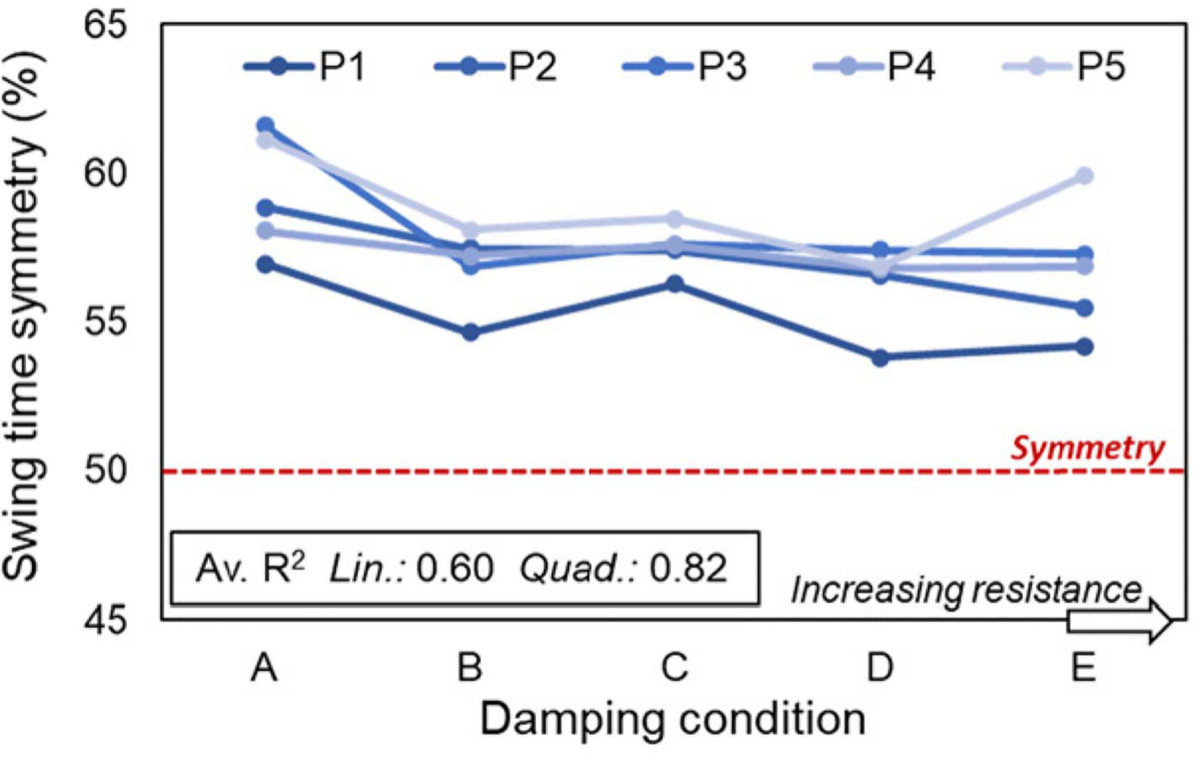
Swing time symmetry. Expressed as 100 * prosthetic swing time / (prosthetic side swing time + sound side swing time). 50% represents symmetry; 50–100% indicates prosthetic swing phase duration is greater than sound side swing phase. A-E in order of increasing damping. Each line represents a different participant (P1-P5) with each point the average (mean) of 5 trials. Av, R^2^ indicates linear (lin) and quadratic (quad) goodness-of-fit, computed on an individual basis with respect to KFD_SW_ and averaged across participants.

All variables exhibited clear trends with increasing KFD_SW_, with approximations on average improving in all cases using a 2nd order polynomial (quadratic) fit (Figs. 2–4), largely due to plateauing at higher KFD_SW_ as knee flexion tended towards zero.

In support of our expectation, the reduction in effective leg length during early swing was progressively attenuated with increasing damping (ELL_MIN_; Fig. 2a, b). In other words, increasing knee flexion damping lead to the maintenance of a more extended prosthetic leg during swing, with all other prosthetic factors held constant.

### Effect of Swing Phase Knee Flexion Damping on Toe Clearance (Q1)

Minimum toe clearance reduced with increased damping (Fig. 2c, d; note negative values reflect toe approximation). This was particularly evident in participants 3–5 who exhibited a clear systematic decrease in toe clearance (see Fig. 2d for representative data from individual participant P3).

### B. Effect of Swing Phase Knee Flexion Damping on Timing of Key Instances (Q2)

The timing at which the limb reached its greatest effective length, ELL_MAX_t shifted progressively earlier in the swing phase with increasing KFD_SW_ (Fig. 3a); closer to the timing of mid swing (Fig. 3b). The timing of minimum toe clearance, TC_MIN_t occurred progressively earlier in swing with increasing KFD_SW_ (Fig. 3c) and transitioned from occurring after mid swing at lower KFD_SW_ to before mid-swing at higher KFD_SW_ (Fig. 3d). Similarly, maximum knee extension timing, KE_MAX_t, occurred progressively earlier in swing phase with increasing KFD_SW_ (Fig. 3e, f).

There was a relatively longer swing phase on the prosthetic limb in comparison to the sound limb, however swing time asymmetry was highest in all cases with no fluid damping (condition A). Linear and quadratic regressions exhibited the lowest R^2^ values on average for this variable although in four participants (P1-P4) there was an apparent trend of increasing symmetry with increasing KFD_SW_ (Fig. 4).

### C. Effect of Swing Phase Knee Flexion Damping on the Control of Toe Clearance (Q3)

**Fig. 5.**
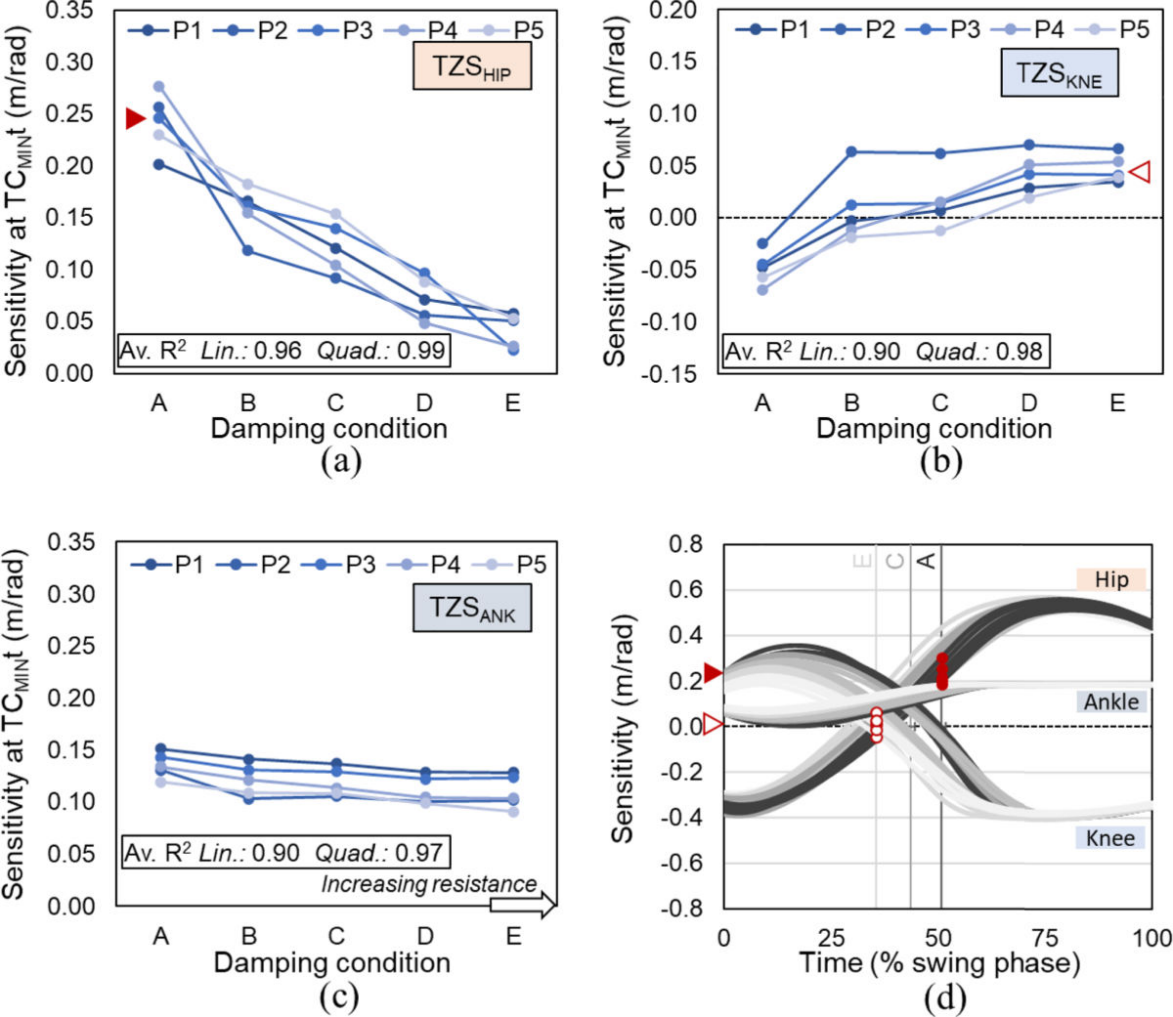
Effect of swing knee flexion damping, KFD_SW_, on vertical toe position sensitivity to lower limb joint rotations, TZS, at the instance of minimum toe clearance, TC_MIN_t. (a) hip, (b) knee and (c) ankle. Positive values indicate clearance of the toe will by increased by joint flexion. (a)–(c) – average (mean) of 5 strides for each participant (P1-P5) at increasing KFD_SW_ levels (A-E). Av, R^2^ indicates linear (lin) and quadratic (quad) goodness-of-fit, computed on an individual basis with respect to KFD_SW_ and averaged across participants. (d) – representative time series data from a single participant (P3) as a function of time, normalized to swing phase; shading from dark to light for three KFD_SW_ conditions (A, C, E) in order of increasing damping. Vertical lines indicate average TC_MIN_t for each damping condition. Red arrows (a, d) illustrate derivation of a two averaged TZS points from individual data (P3) at the lowest and highest damping conditions (A and E).

TZS_HIP_ was positive in all damping conditions, indicating that toe clearance would increase at its lowest point with an increase in hip flexion. The magnitude decreased with increasing KFD_SW_, implying that the vertical toe position was most sensitive to changes in hip flexion at the lowest damping condition (Fig. 5a). Negative TZS_KNE_ values indicated that toe clearance was sensitive to knee extension at lower KFD_SW_, i.e., increases in knee extension would lead to increases in toe vertical position. However, with increasing KFD_SW_ the sensitivity decreased, and the vertical position of the toe at TC_MIN_t became progressively more sensitive to knee flexion (Fig. 5b). Toe position increased with ankle dorsiflexion at all KFD_SW_, illustrated by a positive TZS_ANK_ but became less sensitive to changes at higher KFD_SW_ (Fig. 5c), although changes were modest in comparison to those observed at the hip and knee joints (Fig. 5d).

### D. Effect of Swing Phase Knee Flexion Damping on the Adoption of Compensatory Strategies

**Fig. 6.**
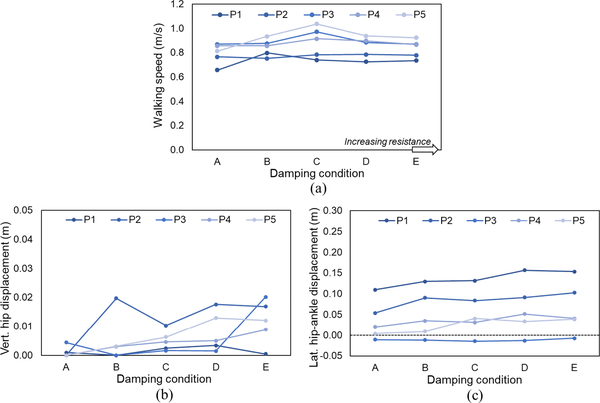
Movement compensations with changes in swing phase knee flexion damping, KFD_SW_. (a) Walking speed. (b) vertical position of hip at instance of minimum toe clearance, expressed as displacement from lowest value across all KFD_SW_ levels. (c) Lateral distance between ankle and hip at instance of minimum toe clearance. Average (mean) of 5 strides for each participant (P1-P5) at increasing KFD_SW_ levels (A-E).

Changes in walking speed, hip vertical displacement strategies and lateral displacement were observed in response to increasing KFD_SW_. The extent varied across participants and damping conditions (Fig. 6). The range of walking speeds was at smallest 0.03 m/s and greatest 0.22 m/s (Fig. 6a; P2, P5 respectively). For hip vertical displacement at TC_MIN_t, two participants (P4, P5) demonstrated a steady increase with increasing KFD_SW_, one increased abruptly only in the highest damping condition (P3) and one adopted a clearance mechanism in the second damping condition that was then attenuated at higher KFD_SW_ (P2; Fig. 6b). Increases in lateral hip-ankle displacement provided evidence of increased limb circumduction with higher KFD_SW_ in four participants (all but P3; Fig. 6c).

## IV. DISCUSSION

We employed a systematic approach to investigate, in isolation of other prosthetic knee design features, the effect of knee flexion damping (KFD_SW_*)* on the swing phase dynamics of transfemoral prosthetic gait. All five participants were experienced prosthesis users and were able to rapidly adapt their movement in order to be able to walk under a range of knee flexion damping conditions.

As predicted, a higher resistance to flexion in early swing resulted in a more limited shortening of the limb in early to mid-swing, reflected by a greater minimum effective leg length (ELL_MIN_, Fig. 2a, b). Further, the time point at which the limb subsequently extended to its greatest length (ELL_MAX_t) occurred earlier in the swing phase, and closer to mid swing; the critical time point at which the swing limb must be shortened sufficiently to allow it to pass the stance limb (Fig. 3a, b).

The consequence of this lack of limb shortening and alteration in timing was a reduction in minimum toe clearance (TC_MIN_*)* with increased damping (Fig. 2c, d). This reduction was observed regardless of the presence of compensatory motions that would act to increase this distance, the extent of which differed across participants and conditions, i.e., without compensation the reduction in TC_MIN_ at higher KFD_SW_ would have been greater. A reduction in TC_MIN_ diminishes the tolerance within which the vertical trajectory of the foot may vary during swing without contacting the ground, and may lead to an increased likelihood of toe catch [21]. Indeed, anecdotally, foot scuff was audible during occasional or frequent strides in the higher damping conditions, although this was not formally recorded.

TC_MIN_ occurred progressively earlier in the swing phase with higher KFD_SW_(Fig. 3c), and its timing transitioned from after to before mid-swing (Fig. 3d). A similar transition with increasing damping is observed in the sensitivity of the vertical toe position to changes in knee angle, TZS_KNE_; from a state at which knee extension will increase toe clearance to one in which knee extension will decrease toe clearance (Fig. 5b). As the prosthetic knee joint is extending non-volitionally at the time of TC_MIN_, the likelihood of a toe catch may therefore be increased at higher KFD_SW_.

Further, the timing of TC_MIN_ may have implications for trip recovery should a toe catch occur when the foot is at its lowest point. A more posterior position of the tripped swing limb with respect to the advancing center of mass of the body will alter the availability of effective strategies for arresting angular momentum generated by such an event. Predominant recovery strategies at earlier time points within the gait cycle are known to differ in transfemoral prosthesis users [22]. Whereas an unimpaired individual might readily raise the tripped foot and place it beyond the obstacle (*elevating strategy* [23]), this is rarely observed on the prosthetic side of individuals with transfemoral amputation [22], likely because it requires the knee to rapidly flex and extend, which is not possible to orchestrate using a conventional knee in the absence of volitional or logic-based control. In contrast, events later in swing may be effectively managed on the prosthetic side in a similar manner to an unimpaired limb; by lowering the foot on the nearside of the obstacle [22], and regulating angular momentum using the ensuing contralateral step [24], providing that an adequate extension moment can be achieved on loading.

The changes in sensitivity of TC_MIN_ to each of the lower limb joints (Fig. 5) reflects differences in control capacity and may also have safety consequences. Given that the hip is the only natural lower limb joint on the affected side, the lesser ability to alter toe clearance via hip angular rotation observed at higher KFD_SW_ (Fig. 5a) could be considered from two opposing perspectives. First, the height of the toe at its lowest point would be less prone to slight changes in hip orientation. Higher KFD_SW_ may therefore offer a greater lenience to user movement variability when considering the small toe clearance tolerance. Second, and conversely, higher damping would provide the user less ability to manipulate the trajectory of the foot should context require it, e.g., when walking on uneven ground, or during trip recovery. The concomitant absence of direct control of the knee and ankle joints may require the adoption or exaggeration of other compensatory strategies such as vaulting and hip hiking to achieve this motor flexibility. Of relevance to the design of combined ankle-knee units, although the sensitivity of vertical toe position to ankle rotation was relatively unaltered with changes in KFD_SW_ (Fig. 5c), its influence per radian of rotation was greater than that of both the hip and the knee at higher damping levels (Fig. 5d). Therefore, should ankle dorsiflexion be timed correctly (noting that TC_MIN_ occurs earlier with increased damping) it could be highly beneficial for reducing the likelihood of toe catch, particularly with higher KFD_SW_. Further, the interplay between KFD_SW_ and joint sensitivity illustrates the effects of functional changes on control strategies, for consideration in prosthetic knee-foot-ankle design.

Whereas the above findings may, on balance, promote selection of a lower KFD_SW_ due to its effect on toe clearance, the opposite is true when considering the requirement for full extension in terminal swing to mitigate risk of the limb buckling due to a flexion moment on loading. The timing of knee extension (KE_MAX_t; Fig. 3e, f) occurred later with lower damping, causing participants to extend the duration of prosthetic swing phase in order to accommodate the delay. With the user’s limited control over the action of the knee after the foot has left the ground, this extension effectively prolongs the proportion of the stride during which there is a diminished ability to readily adjust movement; any attempts to vary cadence or rapidly respond to perturbations may lead to missteps (e.g., loading a flexed limb). Further, in addition to being less desirable from an aesthetics perspective, [14], [17] the induced swing time asymmetry (Fig. 4) may place greater burden on the sound limb for support and control during stance, thus compromising musculoskeletal health [25].

Although these results were obtained using a purely mechanical device, they illustrate important considerations for the adjustment of KFD_SW_ settings that are of relevance to the design of microprocessor-controlled devices. For the motion of the prosthetic knee joint to facilitate locomotion, it must be appropriate given the coordinated movements of the other joints of the body. Acknowledging the holistic effect of different damping settings may be particularly important given the absence of volitional ankle motion that could normally aid limb clearance, and therefore the replication of normative knee dynamics may not necessarily be optimal. For example, despite the timing of TC_MIN_ appearing comparable to normative values when a moderate damping setting is selected (e.g., 45% [26], 51% [18]), the smaller clearance coupled with a potential reduction in ability to recover from a toe catch at this time point might discourage the selection of resistance based purely on this finding. We hope that these insights may contribute towards promising work in algorithm design and optimization protocols that currently trend towards the replication of normative or sound side knee angles [27]–[29].

This study involved a small number of participants, and reports only the acute effect of changes in KFD_SW_ for a novel device. Despite these limitations, clear trends were observed in our variables of interest, several yielding high R^2^ values, and were remarkably consistent across participants even in the presence of individual movement compensations. This consistency may be owing to a number of factors related to the study cohort and design. All participants were experienced prosthesis users and demonstrated an ability to operate the knee effectively prior to testing. Although this may limit the generalizability of results, it permitted testing of the independent variable of interest, KFD_SW_, and did not compromise safety. TC_MIN_ and STS produced the lowest R^2^ values on average; unsurprising as they arguably would be most affected by compensatory movements of the individual rather than device behavior alone.

Participants were permitted limited acclimation time both to the device and to each specific test condition, although the test session may be considered comparable to a clinical scenario during which a limb may be fitted and adjusted during the same appointment. That four of five participants were accustomed to using microprocessor-controlled devices may have further increased the requirement for acclimation. Acclimation was not quantified and comparison with any reported acclimation periods empirically tested in other individuals (see e.g., [30] for technique and sample data) would not be appropriate. However, the trends in the variables we report, which were evident regardless of the adoption of compensatory strategies that varied across participants and across conditions, lend weight to our findings. Further of note, the participant who did not attend a preliminary session revealed a similar pattern of results to those who did, despite omitting bouts of treadmill walking in each damping condition that afforded several hundred steps to aid familiarization. It is therefore plausible that a greater acclimation time would lead to a reduction in the small amount of variability observed on a case-by-case basis and clearer trends evident in higher R^2^ values, rather than refuting the findings of this study. For example, attenuation of the abrupt and excessive increase in hip vertical position of P2 in condition B might be reflected in a clearer trend in the TC_MIN_ for this participant. However, the possibility that greater opportunity to acclimate could also lead to the adoption of fundamentally different movement strategies cannot be discounted. An influence of order bias is similarly possible.

In order to analyze more natural responses to changes in KFD_SW_ we did not prescribe walking speed. In addition to reflecting compensatory behavior when compared across damping conditions, differences observed in walking speed in comparison to habitual gait could reflect limitations of the prototype device. Three of the five individuals produced walking speeds at maximum that were equal to, or within 5% of, their walking speed with their own prosthesis, providing confidence that the prototype device itself permitted gait that was functionally close to habitual locomotion. Of note, two of these individuals were microprocessor knee users. The remaining participants (P3, P4) were the two who normally walked the fastest. It is conceivable that the reduction in speed may be due to a lack of the augmented resistance that would be produced by their own devices at these higher velocities, and at greater knee flexion angles; *heel rise* became excessive, constraining speed. This is consistent with the higher TC_MIN_ values attained for these two participants at the lowest two damping settings, and provides further evidence in favor of second order damping, i.e., resistance proportional to square of angular velocity, or mechanisms with a similar action at higher velocities [31]. This finding has important implications when considering the requirement to walk at different speeds in daily life. We anticipate that the assessment of the effect of KFD_SW_ on stride dynamics at different prescribed walking speeds would reveal further movement compensations of relevance to safety and efficiency. Similarly, with simple first order damping as a baseline, emulating and exploring the effect of the more adaptive resistance profiles of several modern knee units will be an important aim for future empirical and simulation studies.

The rigid body assumption used in the approximation of toe position precluded the calculation of similar variables on the sound limb, and direct comparisons with able-bodied data, due to the possibility of mid-foot movement of a natural foot during the swing phase. The within-subjects design will lessen the impact of error in prediction of the physical location, however absolute magnitudes should be interpreted with caution, including comparisons to published values, many of which will similarly have inbuilt assumptions. Further, the application of these results to inform polycentric knee configuration is limited by the greater toe clearance during swing observed with some polycentric knee units, resulting from changes in the instantaneous axis of rotation across the gait cycle [2].

## V. CONCLUSION

Our results in a small group of individuals with transfemoral amputation demonstrate the implications of altering prosthetic KFD_SW_ on limb dynamics, as summarized in Table II. Lower resistance to flexion tends to increase clearance of the foot and result in a later timing of the minimum value with respect to the swing phase, which may facilitate recovery should a trip event simultaneously occur. Higher damping reduces prosthetic swing duration, promoting a more symmetrical movement. The vertical height of the toe is more sensitive to hip rotation at the time of minimum toe clearance in lower in contrast to higher damping settings. These results may further aid in the configuration of passive prostheses and inform the design of devices under algorithmic control. Future work combining outcomes from similar *in-vivo* studies with numerical simulation may permit the identification of solution spaces while accounting for individual user motor responses.

## Supplementary Material

supp2-3082459

supp1-3082459

## Figures and Tables

**TABLE I:** Participant Information and Selected Damping Conditions (A-E)

#	M/F	Age (yrs)	Ht (m)	Mass (kg)	Amp Side	Et	Time PU (yrs)	Av WS (m/s)	KFD_sw_:Damping coefficients (Nm/(rad/s))				
									A	B	C	D	E
1	M	62	1.79	84.3	L	Tr	50	0.84	0	0.56	0.80	1.32	1.44
2	M	67	1.67	110.8	L	Tr	12	0.78	0	0.56	0.80	1.32	1.44
3	F	51	1.66	68.7	R	Tr	29	1.24	0	0.37	0.56	0.80	1.10
4	F	27	1.53	54.9	R	Ca	25	1.36	0	0.37	0.56	0.80	1.10
5	F	30	1.49	49.4	R	Ca	20	1.05	0	0.37	0.56	0.80	1.10

Ht = Height; Amp. = Amputation; Et = Etiology; Tr = Trauma; Ca = Cancer; Time PU = Duration as a prosthesis user; Av WS = Average walking speed

**TABLE II:** Summary of Findings

Lower resistance	Higher resistance
• Delayed extension in terminal swing. • Promotes prolonged swing phase duration on prosthetic side at the expense of symmetry.	• Shorter swing phase on prosthetic side, promoting symmetry.
• Greater toe clearance.	• Reduced toe clearance.
• Later, post mid-swing, timing of minimum toe clearance.	• Earlier, pre mid-swing timing of minimum toe clearance.
• Greater sensitivity of toe height to hip movement at time of minimum clearance.	• Lesser sensitivity of toe height to hip movement at time of minimum clearance

Effects of swing phase knee flexion damping at lower and higher settings, in five experienced transfemoral prosthesis users walking with a mechanical uniaxial knee.
