# Supplementary material for: Knee Swing Phase Flexion Resistance Affects Several Key Features of Leg Swing Important to Safe Transfemoral Prosthetic Gait
Source: IEEE Trans Neural Syst Rehabil Eng. Author manuscript; Available in PMC 2021 Jun 24. (PMC8223905; doi:10.1109/TNSRE.2021.3082459)
Supplement: supp2-3082459 [file NIHMS1711343-supplement-supp2-3082459.docx]

Knee and hip angles at TC_MIN_t


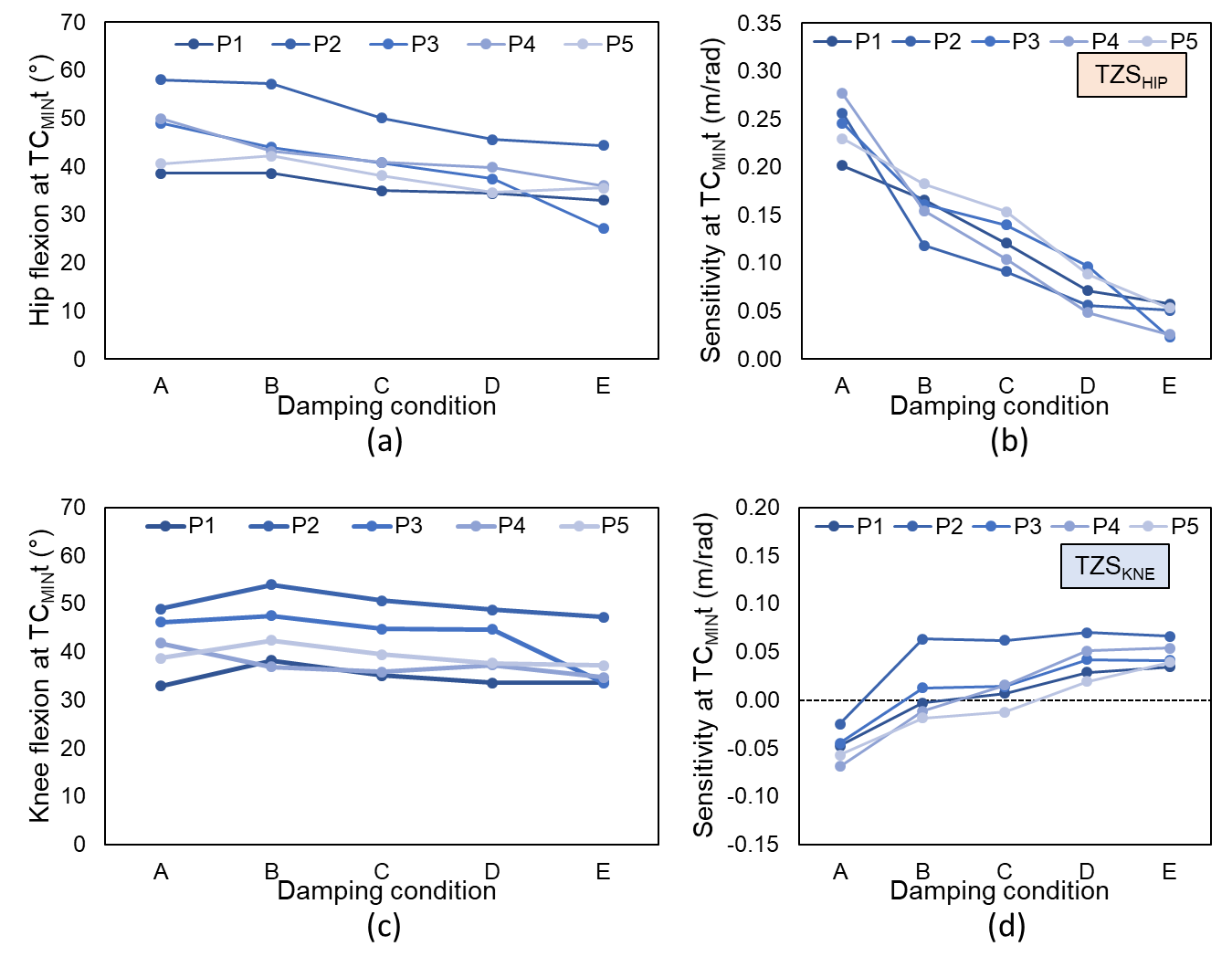


Effect of swing knee flexion damping, KFD_SW_, at the instance of minimum toe clearance, TC_MIN_t, on *(left)* hip (a) and knee (c) flexion angles, and *(right)* vertical toe position sensitivity (TZS) to (b) hip joint rotation, (d) knee joint rotation.

For (b),(d), positive values indicate clearance of the toe will by increased by joint flexion. Average (mean) of 5 strides for each participant (P1-P5) at increasing KFD_SW_ levels (A-E). Note (b), (d) are identical to main text.
