# Supplementary material for: Knee Swing Phase Flexion Resistance Affects Several Key Features of Leg Swing Important to Safe Transfemoral Prosthetic Gait
Source: IEEE Trans Neural Syst Rehabil Eng. Author manuscript; Available in PMC 2021 Jun 24. (PMC8223905; doi:10.1109/TNSRE.2021.3082459)
Supplement: supp1-3082459 [file NIHMS1711343-supplement-supp1-3082459.docx]

Biomechanical Protocol Description

[Fundamentals 2](#_Toc35506975)

[System 2](#_Toc35506976)

[Processing 2](#_Toc35506977)

[Model 2](#_Toc35506978)

[Model description 3](#_Toc35506979)

[Landmarks 3](#_Toc35506980)

[Segment definitions 5](#_Toc35506981)

# Fundamentals

## System

| Capture system / software | 12-camera, Motion Analysis Corporation / Cortex version 7.xxx ^[[1]](#footnote-1)^ |
| --- | --- |
| Medium | Passive retro-reflective markers : 12.5mm with ~2mm fabric/velcro base |
| Sampling frequency | 120Hz |

## Processing

|  | Software | Details |
| --- | --- | --- |
| Eventing | Visual 3D^[[2]](#footnote-2)^ | Force plate threshold (5N) plus Target Pattern Recognition (TPR) for event following force plate contact^[[3]](#footnote-3)^. |
| Filtering | Cortex1 | Kinematic data: 6Hz |
| Interpolation | Cortex1 | Cubic spline / software-based virtual join1 |

## Model

| Design | Skin-mounted, marker configuration based on Orthotrak full body model, modified Helen Hayes version^[[4]](#footnote-4)^ |
| --- | --- |
| Degrees of freedom | 6, with common markers. |
| Optimisation | Segment optimisation - Software generic. See Visual 3D documentation^[[5]](#footnote-5)^ |

# Model description

## Landmarks

Landmarks in parentheses indicate markers that were applied but not used for the present analysis.

* Marker removed following static calibration trial

| Landmark ID  (^bilateral/side) | Location | Location method | Function | | Type |
| --- | --- | --- | --- | --- | --- |
|  |  |  | Definition | Tracking |  |
| ^ASI | Superficial on skin surface such that marker body is anterior to prominent anterior edge of iliac crest. | Palpation | Yes | Yes | Marker |
| VSAC | Superficial to the spine at approximately L5 level. | Palpation | Yes | Yes | Marker |
| (LPSI) | Superficial to palpable prominence at posterior edge of iliac crest. Legacy labelling marker. | Palpation | No | No | Marker |
| ^HJC | Hip joint centre – offset from pelvis origin based on pelvis depth (anterior-posterior distance between ASIs and VSAC), pelvis width (inter-ASIS distance) and leg length, according to Harrington et al.^[[6]](#footnote-6)^ | Calculation | Yes | Yes | Derived |
| ^THI | Anteriorly on lower thigh, at approximately 1/3 thigh length. | Visualisation | No | Yes | Marker |
| ^KNE | Superficial to the lateral femoral condyle at the knee flexion/extension axis of rotation. | Visualisation | Yes | Yes | Marker |
| ^KNE_2 | Superficial to the medial femoral condyle, to form the medial end of the knee flexion/extension axis with ^KNE. | Visualisation | Yes | No | Marker* |
| ^VKNL | KNE projected towards KNE_2 a distance equal to marker radius. | Calculation | Yes | No | Derived |
| ^VKNM | KNE_2 projected towards KNE a distance equal to marker radius. | Calculation | Yes | No | Derived |
| ^KJC | Midpoint between KNE and KNE_2. | Calculation | Yes | No | Derived |
| ^SHA | Anteriorly on shank, approximately 1/3 distance from proximal to distal end. | Visualisation | No | Yes | Marker |
| ^ANK | At apex of lateral malleolus, to form the lateral end of the ‘ankle’ axis with ^ANK_2. | Palpation | Yes | Yes | Marker |
| ^ANK_2 | At apex of medial malleolus, to form the medial end of the ‘ankle’ axis with ^ANK. | Palpation | Yes | No | Marker^*^ |
| ^VANL | ANK projected towards ANK_2 a distance equal to marker radius. | Calculation | Yes | No | Derived |
| ^VANM | ANK_2 projected towards ANK a distance equal to marker radius. | Calculation | Yes | No | Derived |
| ^.TO | At location approximating point between 2^nd^ and 3^rd^ metatarsal heads, on dorsum of shoe. | Visualisation | Yes | Yes | Marker |
| ^.ME | On dorsum of foot, proximal and lateral to .TO. | Visualisation | No | Yes | Marker |
| ^HEE | On heel counter, aligned vertically with .TO, to form the longitudinal axis of the foot with .TO | Measurement /Visualisation | Yes | Yes | Marker |
| ^TOEvert | .TO marker projected vertically onto plane of laboratory floor. | Calculation | Yes | No | Derived |
| ^HEEvert | HEE marker projected vertically onto plane of laboratory floor. | Calculation | Yes | No | Derived |
| ^HEElat | HEEvert marker projected laterally by 0.1m | Calculation | Yes | No | Derived |
| ^VDFT | Interim distal foot landmark – projected from HEEvert parallel to line connecting HEEvert and TOEvert a distance equal to measured foot length plus marker radius. | Calculation | Yes | No | Derived |
| ^VEXT  or ‘vTOE’ | VDFT established within coordinate system of foot to permit tracking during movement. | Calculation | No | Yes | Derived |
| (^SHO) | Superficial to and vertically above the acromium process. | Palpation | Yes | Yes | Marker |
| (SCAP) | Superficial to the right scapula. Legacy labelling marker. | Visualisation | No | No | Marker |
| (^ELB) | Superficial to the lateral epicondyle, placed with the arm flexed to 90°. | Palpation | Yes | Yes | Marker |
| (^WRI) | Midpoint between the radial and ulnar prominences on the dorsal wrist. | Visualisation | Yes | Yes | Marker |

## Segment definitions

| Segment (^bilateral/  side) | Landmarks (derived landmarks in parentheses) | Origin | Axes | | | Tracking markers |
| --- | --- | --- | --- | --- | --- | --- |
|  |  |  | Flex/ext | Add/abd | Axial |  |
| ^ Thigh | (HJC), (VKNL), (VKNM), THI | HJC | Perpendicular to axial axis in plane defined by HJC, VKNL & VKNM | Orthogonal to axial and flex/ext axes | Line joining HJC and midpoint between VKNL & VKNM | HJC, THI, KNE |
| ^ Shank | (KJC), (VANL), (VANM), KNE, SHA | KJC | Perpendicular to axial axis in plane defined by KJC, VANL & VANM | Orthogonal to Axial and Flex/Ext axes | Line joining KJC and midpoint between VANL & VANM | KNE, SHA, ANK |
| ^ Foot | (HEEvert), (TOEvert), (HEElat), .ME, .TO, HEE | AJC | Perpendicular to Add/abd axis in plane defined by HEEvert, TOEvert and HEElat | Line joining HEEvert and TOEvert | Orthogonal to Axial and Flex/Ext axes | .TO, HEE, /ME |

1. Motion Analysis Corporation, Santa Rosa, CA, USA. [↑](#footnote-ref-1)
2. C-Motion, Germantown, MD, USA. [↑](#footnote-ref-2)
3. <http://c-motion.com/v3dwiki/index.php?title=Event_TPR_Signal> [↑](#footnote-ref-3)
4. Motion Analysis Corporation (2009). Joint Angles and Segment Coordinate Systems. Orthotrak Version 6.6 Reference Manual, H4-H18. [↑](#footnote-ref-4)
5. <http://www.c-motion.com/v3dwiki/index.php?title=Six_Degrees_of_Freedom> [↑](#footnote-ref-5)
6. Harrington ME, Zavatsky AB, Lawson SE, Yuan Z, Theologis TN.(2007) Prediction of the hip joint centre in adults, children, and patients with cerebral palsy based on magnetic resonance imaging. J Biomech. 2007;40(3):595-602. Epub 2006 Apr 3. [↑](#footnote-ref-6)
